# Alterations in serum levels of copper, zinc, and selenium among children with sickle cell anemia

**DOI:** 10.3906/sag-1812-92

**Published:** 2019-10-24

**Authors:** Rana HASANATO

**Affiliations:** 1 Department of Pathology, College of Medicine, King Saud University, Riyadh Kingdom of Saudi Arabia

**Keywords:** Sickle cell anemia, trace elements, copper, zinc, selenium

## Abstract

**Background/aim:**

Oxidative stress contributes to pathophysiological dysfunction in sickle cell anemia (SCA). Copper (Cu) is a prooxidant, whereas zinc (Zn) and selenium (Se) are antioxidant trace elements. This study investigates the serum levels of Cu, Zn, and Se among children with SCA.

**Materials and methods:**

This cross-sectional study was performed at King Khalid University Hospital, Riyadh. Thirty-three children with SCA in steady state and 33 age- and sex-matched normal healthy children were included in the study. Cu, Zn, and Se levels were measured by inductively coupled plasma-mass spectrometry (ICP-MS) instrument.

**Results:**

The median serum Cu levels among SCA patients (1.3 μg/mL) were higher than those of the controls (0.88 μg/mL; P < 0.0001). Zn (0.61 μg/mL) and Se (74 ng/mL) levels among SCA patients, however, were significantly lower than those of the controls (0.94 μg/mL; P < 0.0001) and (91.2 ng/mL; P < 0.0001), respectively. The Cu/Zn ratio among SCA patients (1.92) was higher than that of the controls (0.98).

**Conclusion:**

Decreased blood levels of antioxidant trace elements may contribute to the pathophysiology in SCA by promoting oxidative stress. The monitoring of trace element levels in SCA appears to be vital for decreasing morbidity associated with the disorder.

## 1. Introduction

Sickle cell anemia (SCA) is a hemoglobinopathy that manifests in infancy and is associated with shortened life expectancy due to repeated episodes of acute illness and organ damage [1]. It is characterized by a predominant presence of sickle hemoglobin (HbS) resulting from a substitution of glutamic acid to valine at sixth amino acid position in the β globin chain [2]. HbS is inherently unstable and tends to polymerize under low oxygen tension resulting in sickling of red blood cells, hemolysis, and anemia [3]. Sickling and hemolysis in SCA are associated with a high level of oxidative stress due to increased production of superoxide anion, reactive oxygen species (ROS), and impaired activity of ROS-scavenging enzymes [4]. Low level of ROS activity has been attributed to inadequate vitamin intake or decreased production of enzymes due to cofactors deficiency [5].

Copper (Cu) and zinc (Zn) as cofactors are essential for the optimal performance of superoxide dismutase (SOD), an ROS scavenging enzyme responsible for handling superoxide radicals, thus preventing tissue damage [6,7]. Zn as a trace element behaves as antioxidant [8], whereas Cu is a prooxidant [9]. SCA patients are more susceptible to alterations in these trace element concentrations because of preexisting oxidative stress due to accelerated autooxidation of HbS [10]. Selenium (Se) is essential for a wide variety of cellular functions. Human selenoprotenome comprised of 25 separate genes encodes for selenoproteins, including glutathione peroxidase, a member of ROS scavenging enzymes [11]. Se acts as an antioxidant and plays a crucial role in the defense against oxidative stress [12]. Although a number of investigations have assessed oxidative stress in SCA, the data regarding the status of anti- and prooxidant trace elements among patients with SCA are insufficient, particularly in the Kingdom of Saudi Arabia. This study was performed at King Khalid University Hospital, Riyadh, with the aim to assess serum levels of Cu, Zn, and Se among children with SCA in steady state.

## 2. Materials and methods

This cross-sectional study was performed in the Clinical Chemistry Unit at King Khalid University Hospital, Riyadh, between July 2015 and September 2016. A total of 33 patients with SCA were recruited from the hematology clinic at King Khalid University Hospital, Riyadh, Kingdom of Saudi Arabia. The diagnosis of SCA was confirmed by hemoglobin electrophoresis. Prior to enrollment in the study, it was ensured that none of the participants was receiving trace element supplements either at the time of enrollment or at least three months prior. Any patient with β- or α-thalassemia trait, G6PD deficiency, receiving treatment with hydroxyurea, receiving blood transfusion less than one month prior to enrollment, and presence of any chronic illness other than the primary disease was excluded from the study. After obtaining informed consent, 5 mL of venous blood were collected aseptically in a vacutainer without anticoagulant. Serum was separated from each sample by centrifugation and was stored at –80 °C until used. Cu, Zn, and Se levels were measured by inductively coupled plasma-mass spectrometry (ICP-MS) instrument (Perkin-Elmer, Warsaw, Poland). This study was approved by the Institutional Review Board (IRB) of the College of Medicine, King Saud University, Riyadh, Kingdom of Saudi Arabia. Reference number: 15/0292/IRB. Project number: E-15-1380. All participants/parents provided informed consent prior to inclusion in the study.

### 2.1. Statistical analysis

Data were analyzed by MedCalc computer software, version 14.8.1. Categorical data were summarized as numbers and percentages. Numeric data were summarized as means and standard deviations, or medians and interquartile ranges. Comparison between groups for categorical markers was performed using the chi-square test or Fisher’s exact test. Comparisons between groups for continuous variables were done by using independent sample t-test or the Mann–Whitney U test. P < 0.05 was considered statistically significant. 

## 3. Results

**Figure 1:**
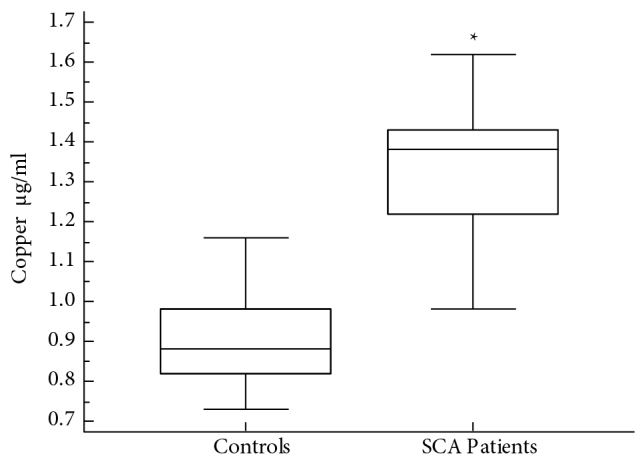
Comparison of serum copper levels between patients with SCA and normal controls. *P < 0.0001

**Figure 2:**
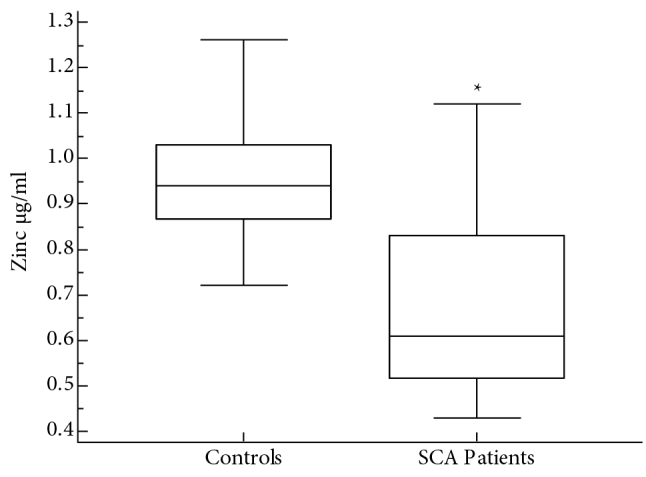
Comparison of serum zinc levels between patients with SCA and normal controls. *P < 0.0001

**Figure 3:**
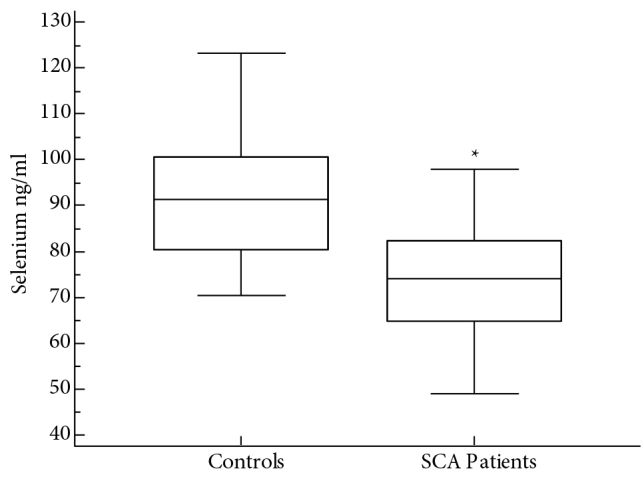
Comparison of serum selenium levels between patients with SCA and normal controls. *P < 0.0001.

Thirty-three children with SCA (19 males and 14 females; mean age 8.5 ± 4.1 years) of consenting parents were included in the study. A group of 33 healthy children (18 males and 15 females; mean age 7.3 ± 3.7 years) was also included in the study as normal controls. Patients with SCA had low mean hemoglobin level of 91.3 ± 8.1 g/L compared to controls (139.7 ± 6.2; P < 0.001). The mean HbS percentage among children with SCA was 86.6 ± 7.1%, whereas none of the controls had HbS. HBA2 among children with SCA (3.4 ± 2.4%) was not different from that of the controls (2.8 ± 1.3%). Mean percentage of HBF (10.1 ± 5%) among children with SCA was higher than controls (1.9 ± 0.8%; P < 0.005) (Table). Data comparing serum levels of Cu between patients with SCA and normal controls are shown in Figure 1. The median serum level of Cu among patients with SCA (1.3 μg/mL) was significantly higher than in normal controls (0.88 μg/mL; P < 0.0001). Figure 2 compares serum levels of Zn between patients with SCA and controls. The median level of Zn among the patients with SCA (0.61 μg/mL) was significantly lower than that in normal controls (0.94 μg/mL; P < 0.0001). Figure 3 shows the data for serum levels of Se among patients with SCA and controls. The median serum level of Se (74 ng/mL) among patients with SCA was significantly lower than that in normal controls (91.2 ng/mL; P < 0.0001). Cu to Zn ratio (Cu/Zn) among the control group was 0.98, whereas Cu/Zn among children with SCA was 1.92.

**Table 1:** Characteristic features of children with sickle cell anemia (SCA) and normal controls.

Parameter	Patients with SCA	Normal controls	P-value
Age (years)	8.5 ± 4.1	7.3 ± 3.7	0.21
Males, n (%)	19 (57.5%)	18 (54.5%)	0.803
Females, n (%)	14 (42.4%)	15 (45.4%)	0.803
Male/female ratio	1.3	1.2	-
Hemoglobin (g/L)	91.3 ± 8.1	139.7 ± 6.2	0.001
Hemoglobin S%	86.6 ± 7.1	None	-
Hemoglobin A2%	3.4 ± 2.4	2.8 ± 1.3	0.09
Hemoglobin F%	10.1 ± 5	1.9 ± 0.8	0.005
Hemoglobin A%	None	97.1 ± 0.7	-
Painful episodes / year	4.3 ± 2.7	None	-
Infections / year	5.1 ± 1.7	2.3 ± 1.7	0.02

Patients with SCA: n = 33; normal controls: n = 33

## 4. Discussion

Children with SCA had elevated serum Cu levels, whereas serum levels of Zn and Se were significantly lower than those in controls. High levels of serum Cu among SCA patients have been reported previously [13]. Free Cu ions promote the production of reactive hydroxy radicals and may therefore contribute to prooxidant activity in SCA [14]. The availability of free Cu ions is facilitated by concentrations of Cu higher than the binding capacity of ceruloplasmin serving as an antioxidant [15]. Increased urinary loses of Zn among patients with SCA [16] may also contribute to higher Cu levels and increased Cu/Zn ratio, as observed in the present study. Moreover, the higher amount of zincuria among SCA patients, by causing Zn deficiency, triggers enhanced Cu absorption from the gastrointestinal tract that may have contributed to elevated serum Cu levels [17]. Collectively, the evidence suggests that among patients with SCA, the Cu and Zn homeostasis is skewed in favor of prooxidant activity.

The serum levels of Zn were significantly lower among SCA patients compared to those of controls in the present study. Elevated levels of copper and low levels of Zn among patients with SCA have been reported previously [18], indicating a compromised status of defense against oxidative stress. Zn supplementation among patients with SCA not only restores antioxidant reserves, but also decreases the incidence of infections with increased hemoglobin and hematocrit levels [19]. Restoration of serum Zn levels not only markedly reduces the tendency for sickling of red blood cells but is also associated with a remarkable reduction in the incidence of vaso-occlusive events among SCA patients [20,21]. Zn deficiency has been implicated in a number of immune abnormalities [22]. Supplementation of Zn in patients with SCA has also been shown to restore impaired delayed type of hypersensitivity reaction and abnormalities in natural killer cell function [19]. The diversity of abnormalities described among SCA patients [22] may be linked directly or indirectly with Zn deficiency, as Zn has been shown to be an essential cofactor for over 300 enzymes [23].

SCA patients in the present study had a higher Cu/Zn ratio compared to normal controls. Increased Cu/Zn ratio has been documented in SCA previously and is considered a useful adjunct for the diagnosis of SCA [24]. Successful treatment of hyperglycemia with resveratrol in type 2 diabetes mellitus is associated with a remarkable reduction in oxidative stress along with decreased Cu/Zn ratio due to the lowering of serum Cu levels [25]. These findings indicate that the assessment of Cu/Zn ratio may serve as a useful tool for gauging oxidative stress. Periodic assessment of Cu/Zn ratio among SCA patients, therefore, appears to be useful not only for monitoring oxidative stress among these patients, but may also preempt an impending sickle cell crisis.

Compared to normal controls, serum levels of Se were significantly lower among SCA patients in the present study. Though infrequently, low blood levels of Se have been reported among patients with SCA [26], suggesting a weak antioxidant potential. The antiinflammatory role of Se was recently demonstrated in an experimental study on adiponectin knockout mice where Se was shown to provide protection against chronic inflammation-induced colon cancer [27]. Selenoproteins are Se containing proteins mediating biological effects of Se [28]. Glutathione peroxidases are selenoproteins and are the main antioxidants involved in neutralizing the production of ROS [29]. Low levels of glutathione peroxidase among patients with SCA [26] highlights the importance of Se in handling the oxidative stress associated with SCA. In addition, Se has been shown to provide protection against oxidative stress-induced lipid peroxidation and disturbances in fatty acid biosynthesis among patients with SCA [30].

In conclusion, children with SCA had low levels of Zn and Se with elevated serum levels of Cu. Decreased blood levels of antioxidant trace elements may contribute to the pathophysiology in SCA by promoting oxidative stress. The monitoring of trace element levels in SCA appears to be important for reduction in disease associated morbidity among children with SCA. Large-scale studies are recommended to validate the findings of this study and further elucidate the role of trace elements in sickle cell disease.

## Acknowledgment

The author would like to thank Mr. Mohammed Atiya for his assistance with the experimental work.
